# Loss of ULK1 Attenuates Cholesterogenic Gene Expression in Mammalian Hepatic Cells

**DOI:** 10.3389/fcell.2020.523550

**Published:** 2020-09-30

**Authors:** Sangam Rajak, Liliana F. Iannucci, Jin Zhou, B. Anjum, Nelson George, Brijesh K. Singh, Sujoy Ghosh, Paul M. Yen, Rohit A. Sinha

**Affiliations:** ^1^Department of Endocrinology, Sanjay Gandhi Postgraduate Institute of Medical Sciences, Lucknow, India; ^2^Program of Cardiovascular and Metabolic Disorders, Duke-NUS Medical School, Singapore, Singapore; ^3^Department of Biology, University of Padua, Padua, Italy

**Keywords:** ULK1, mevalonate pathway, cholesterogenesis, SREBF2, FOXO3A transcription factor

## Abstract

The hepatic mevalonate (MVA) pathway, responsible for cholesterol biosynthesis, is a therapeutically important metabolic pathway in clinical medicine. Using an unbiased transcriptomics approach, we uncover a novel role of Unc-51 like autophagy activating kinase 1 (ULK1) in regulating the expression of the hepatic *de novo* cholesterol biosynthesis/MVA pathway genes. Genetic silencing of ULK1 in non-starved mouse (AML-12) and human (HepG2) hepatic cells as well as in mouse liver followed by transcriptome and pathway analysis revealed that the loss of ULK1 expression led to significant down-regulation of genes involved in the MVA/cholesterol biosynthesis pathway. At a mechanistic level, loss of ULK1 led to decreased expression of SREBF2/SREBP2 (sterol regulatory element binding factor 2) via its effects on AKT-FOXO3a signaling and repression of SREBF2 target genes in the MVA pathway. Our findings, therefore, discover ULK1 as a novel regulator of cholesterol biosynthesis and a possible druggable target for controlling cholesterol-associated pathologies.

## Introduction

Cholesterol, at the physiological level, serves as a major constituent of cellular membranes and a precursor for bile and steroid hormone synthesis (Tabas, 2002). However, the pathological increase in cholesterol levels has been attributed as a leading cause of several cardiovascular and metabolic disorders (Tabas, 2002). Cholesterol can be synthesized *de novo* via the mevalonate (MVA) pathway in liver, and may also be obtained from food sources (Ye and Debose-Boyd, 2011). Interestingly, it is the endogenous synthesis of cholesterol within liver, via the MVA pathway, which has been more closely linked to the development of cardiovascular complications (Ye and Debose-Boyd, 2011). Not surprisingly, statins, which target the endogenous cholesterol/MVA biosynthesis pathways, have been very successful in countering hypercholesterolemia. However, despite the successful use of statin therapy in most of the patients, either alone or in combination with other agents, some individuals may not respond positively to statins (Reiner, 2014). This crisis warrants initiatives to discover novel alternatives and adjunct therapies to counter hypercholesterolemia. While it has been studied mostly in relation to its role in cholesterol synthesis and cardiovascular diseases, the MVA pathway has become a potential therapeutic target for several cancers, autoimmune disorders, atherosclerosis, fatty liver, and Alzheimer’s disease (Buhaescu and Izzedine, 2007).

Unc-51 like autophagy activating kinase 1 (ULK1) is a proximal protein involved in mammalian autophagic signaling (Lin and Hurley, 2016). It is the only autophagy protein, which possess a kinase activity and triggers autophagy in response to both energy and nutrient depletion (Lin and Hurley, 2016). ULK1 phosphorylates several other autophagy proteins such as ATG13 and Beclin to initiate cellular autophagy (Lin and Hurley, 2016). Besides general autophagy, ULK1 also regulates selective autophagic processes such as mitophagy (Sinha et al., 2015). Interestingly, several non-autophagic roles of ULK1 have been recently uncovered, which include involvement in interferon signaling, ER-to–Golgi cargo transport, glycolysis, and immune response (Konno et al., 2013; Joo et al., 2016; Joshi et al., 2016; Li et al., 2016). In this line of evidence, we had recently demonstrated an autophagy-independent action of ULK1 in regulating the transcriptional activity of nuclear receptors involved in hepatic lipid metabolism (Sinha et al., 2017). Owing to its kinase activity and druggability, new inhibitors for ULK1 have been recently developed (Egan et al., 2015), however, their use in metabolic diseases has not been tested.

In this study, we examined the signaling pathways that are regulated by ULK1 using transcriptomics, followed by pathway analysis during the non-starved (basal) state.

Our results uncover a novel role of ULK1 in regulating the expression of genes involved in hepatic MVA/cholesterol biosynthesis pathway via the AKT-FOXO3a (forkhead box O3a) mediated regulation of sterol regulatory element binding factor 2 (SREBF2).

## Results

### Genetic and Pharmacological Inhibition of ULK1 Inhibits the Expression of MVA/Cholesterogenic Genes in Mouse AML-12 Cells

**FIGURE 1:**
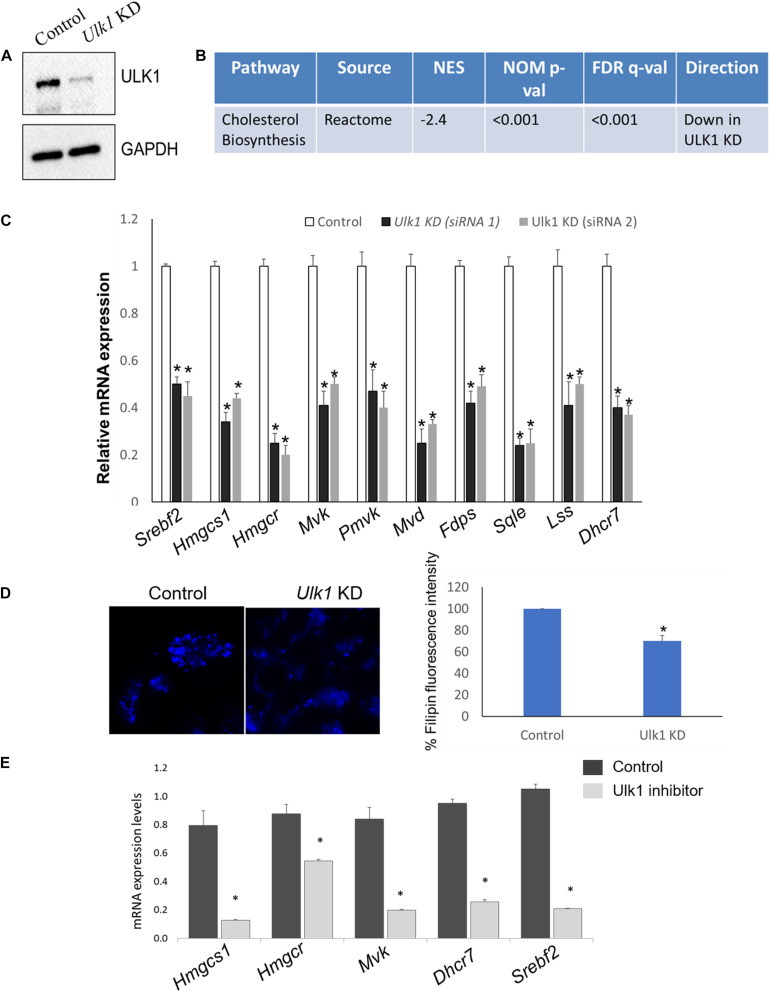
ULK1 regulates mevalonate/cholesterol biosynthesis pathway gene expression in mouse hepatocyte AML12 cells. **(A)** Representative immunoblot showing knockdown (KD) efficiency of ULK1 in AML12 cells treated with ± *Ulk1* siRNA (Thermo Fisher Scientific, s75753 siRNA #1; 10 nM/72 h) or a negative siRNA (denoted as Control). **(B)** Microarray derived transcriptomic pathway analysis in AML12 cells and mouse liver upon *Ulk1* KD as described above. **(C)** qRT-PCR validation of mevalonate/cholesterol biosynthesis pathway genes ± *Ulk1* siRNA’s 1 (Thermo Fisher Scientific, s75753 siRNA #1) and 2 (Thermo Fisher Scientific, s75751 siRNA #2) at 10 nM concentration for 72 h in AML12 cells. Values are means ± SD (*n* = 4), *p < 0.05. **(D)** Filipin staining and quantitation showing intracellular cholesterol levels in AML12 cells treated with ± *Ulk1* siRNA (Thermo Fisher Scientific, s75753 siRNA #1; 10 nM/72 h) or a negative siRNA (denoted as Control). AML12 cells upon *Ulk1* KD (10 nM/72 h) were washed with PBS, fixed, and stained with filipin. All photographs were taken with the same exposure time and 20× magnification. **(E)** qRT-PCR validation of cholesterol biosynthesis genes enriched in the pathway analysis treated with SBI-0206965 (ULK1 inhibitor) at a dose of 10 uM for 48 h in AML12 cells. Values are means ± SD (*n* = 3,^∗^*p* < 0.05).

To identify the signaling pathways affected by ULK1 during nutrient-rich conditions, we silenced *Ulk1* gene in mouse hepatocyte cell line AML-12 using siRNA under non-starved conditions. After confirming knockdown (KD) efficiency of *Ulk1* siRNA (Figure 1A) we performed a microarray analysis of the samples followed by pathway analysis (Supplementary Table S1). Our results showed significant down-regulation of the cholesterol biosynthesis pathway upon loss of ULK1 using the Reactome pathway database (Figure 1B and Supplementary Table S1). Besides the regulation of cholesterol biosynthesis, other closely related pathways such as steroid, terpenoid and triglyceride biosynthesis pathways were also downregulated upon *Ulk1* silencing (Supplementary Table S1).

Of note, the genes involved in the Reactome cholesterol biosynthesis dataset belong to the broader MVA pathway, which is involved in cholesterol biosynthesis within the hepatocytes (Buhaescu and Izzedine, 2007; Supplementary Table S2). Next, we validated the microarray gene using qRT-PCR, showing that the expression of several rate limiting enzymes of the MVA/cholesterol biosynthesis pathway including hydroxymethylglutaryl-CoA synthase 1 (*Hmgcs1*), 3-hydroxy-3-methylglutaryl-CoA reductase (*Hmgcr*) were indeed significantly decreased in the *Ulk1* silenced cells *in vitro* (using two different siRNA) (Figure 1C). Although not present in the gene enrichment dataset, a similar decrease in the expression of the master transcriptional regulator of sterol synthesis, *Srebf2* (Madison, 2016) was also observed upon *Ulk1* silencing (Figure 1C). Furthermore, repression of the cholesterogenic genes upon *Ulk1* KD corroborated with reduced intra-cellular cholesterol in cells (Figure 1D). Additionally, the pharmacological inhibition of ULK1 by a kinase specific inhibitor, SBI-0206965 in AML-12 cells, also exhibited a similar suppression of genes involved in the MVA pathway (Figure 1E) suggesting a role of ULK1 kinase domain in mediating these effects on the MVA pathway genes.

### Genetic Inhibition of ULK1 Inhibits the Expression of MVA/Cholesterogenic Genes in Mouse Liver

**FIGURE 2:**
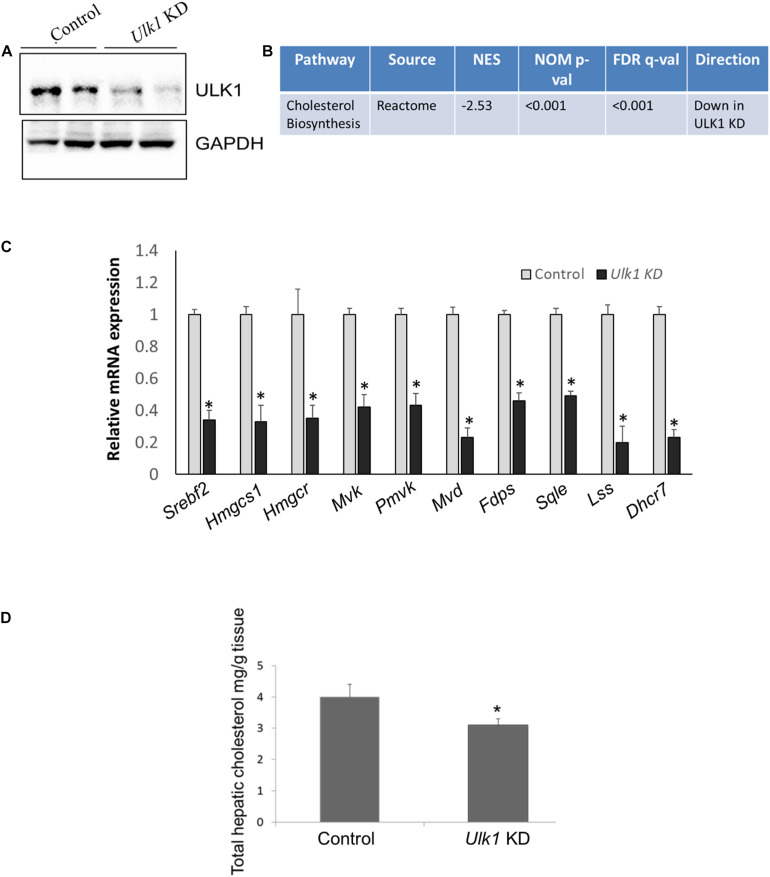
ULK1 regulates mevalonate/cholesterol biosynthesis pathway gene expression in mouse liver. **(A)** Representative immunoblot showing silencing efficiency of ULK1 in mouse liver treated with hydrodynamic tail vein delivered ± *Ulk1* siRNA (40 ug/72 h). **(B)** Microarray derived transcriptomic pathway analysis in mouse liver upon *Ulk1* KD as described above. **(C)** qRT-PCR validation of mevalonate/cholesterol biosynthesis pathway genes ± *Ulk1* siRNA (40 ug/72 h) in AML12 mouse liver. Values are means ± SD (*n* = 4), ^∗^*p* < 0.05. **(D)** Total cholesterol content in hepatic lipid extracts in mouse liver ± *Ulk1* siRNA (40 ug/72 h). Values are means ± SD (*n* = 4, *p < 0.05).

We next validated our *in vitro* data *in vivo*, by silencing *Ulk1* gene in mouse liver using siRNA. In consonance with our *in vitro* results, we observed a similar down-regulation of MVA/cholesterol biosynthesis gene expression upon *Ulk1* silencing in mouse liver using microarray, qRT-PCR and western blotting (Figures 2A–C, Supplementary Figure S1 and Supplementary Tables S3, S4). Additionally, the intra-hepatic cholesterol levels were also reduced marginally yet significantly upon *Ulk1* silencing (Figure 2D). The reduction in serum cholesterol levels, however, did not reach a significant level (Supplementary Figure S2). Therefore, these results confirmed that ULK1 positively regulates MVA/cholesterol biosynthesis genes in mouse liver *in vivo*.

### Loss of *Atg5* and *Becn1* Does Not Alter Cholesterogenic Gene Expression in AML-12 Cells

Next, to clarify the role of autophagy in this process, we observed the effect of siRNA KD of two additional ATG genes, *Atg5* and *Beclin1*/*Becn1*, on the expression of key MVA pathway genes in mouse hepatocyte cells, AML-12. Interestingly, neither *Atg5* nor *Becn1* had any suppressive effect on MVA pathway genes (Supplementary Figure S3) thereby suggesting a probable autophagy-independent action of ULK1 in regulating the expression of these genes.

### Genetic Silencing of ULK1 Inhibits the Expression of MVA/Cholesterogenic Genes in Human Hepatic Cells via AKT-FOXO3a Pathway

**FIGURE 3:**
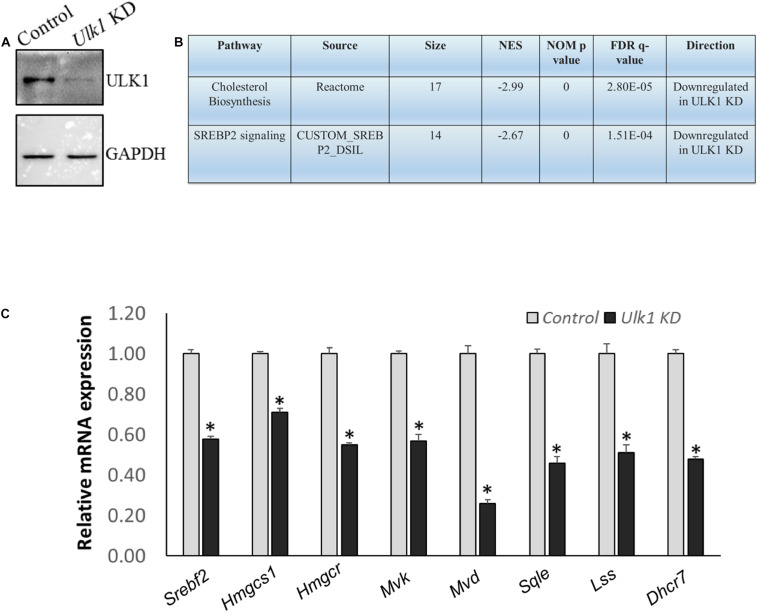
ULK1 loss suppresses the expression of human cholesterogenic genes **(A,B)** Representative immunoblot showing KD efficiency of ULK1 and RNA-seq derived transcriptomic pathway analysis in HepG2 cells treated with ± *Ulk1* siRNA (Thermo Fisher Scientific, s15965; 10 nM/72 h). **(C)** qRT-PCR validation of mevalonate/cholesterol biosynthesis pathway genes ± *Ulk1* siRNA (10 nM/72 h) in HepG2 cells. Values are means ± SD (*n* = 4), ^∗^*p* < 0.05.

We also verified our results obtained from the mouse hepatic cells, in human hepatocytes using HepG2 cells. Using RNA-seq and Real-Time PCR validation analysis, we observed a similar down-regulation of signaling pathways related to human cholesterol biosynthesis and SREBF2 in cells that underwent ULK1 KD (Figures 3A–C and Supplementary Tables S5–S7). Thus, ULK1 regulated the MVA pathway across species in the mammalian hepatic cells.

**FIGURE 4:**
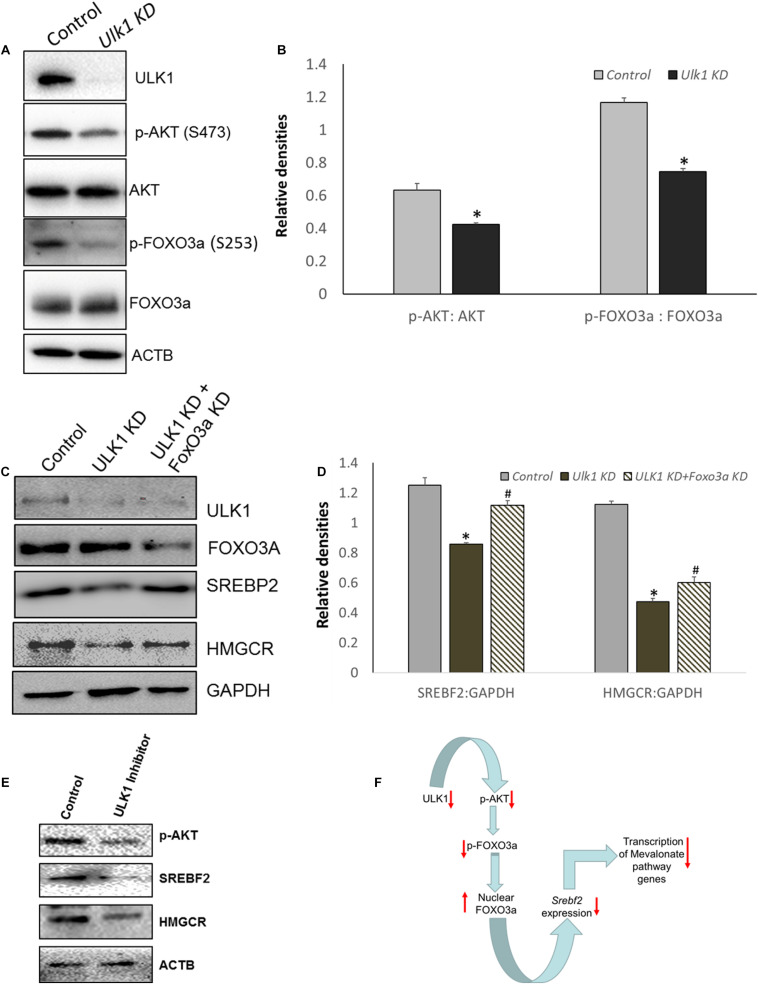
ULK1 loss suppress human cholestrogenic genes via effects on AKT-FOXO3a pathway. **(A,B)** Representative immunoblot and densitometry showing cellular levels of phosphorylated and total AKT and FoxO3a proteins ± *Ulk1* siRNA (10 nM/48 h) in HepG2 cells. Values are means ± SD (*n* = 3),^∗^*p* < 0.05. **(C,D)** Representative Immunoblot and densitometry showing protein levels of SREBF2 and HMGCR in HepG2 ± *Ulk1* alone and with *FoxO3a* (Cell Signaling Technology, #6302) double KD (10 nM) for 48 h of treatment. Values are means ± SD (*n* = 3), ^∗^*p* < 0.05 between *Ulk1* KD vs. Control and #*p* < 0.05 between *Ulk1* KD + *FoxO3a* KD vs. *Ulk1* KD alone. **(E)** Representative immunoblot of HepG2 cells treated with SBI-0206965 (ULK1 inhibitor) at a dose of 10 uM for 48 h. **(F)** Schematic diagram showing the regulation of Mevalonate/cholesterol biosynthesis pathway upon loss of ULK1.

As SREBP2/SREBF2 is the master transcriptional regulator of genes involved in the MVA/cholesterol biosynthesis pathway (Madison, 2016), we further investigated its regulation by ULK1 in HepG2 cells. Anabolic signaling by AKT has been shown to upregulate SREBF2 levels in hepatic cells (Luu et al., 2012). Additionally, AKT regulation of *Srebf2* gene transcription is mediated through AKT induced inhibitory phosphorylation and nuclear exclusion of FOXO3, a transcription factor (Tzivion et al., 2011) that is a transcriptional repressor of *Srebf2* gene (Tao et al., 2013). Therefore, we next investigated whether AKT-FOXO3a signaling was perturbed in the absence of ULK1 in hepatic cells. Interestingly, our results showed that both basal AKT and its substrate FOXO3a phosphorylation was suppressed in hepatic cells in which ULK1 expression was silenced by siRNA (Figures 4A,B). Similar effects were also observed *in vivo* (Supplementary Figures S4A,B). These results strongly suggest that the activation of FOXO3a is a possible mechanism for the *Srebf2* suppression observed in ULK1 KD cells. Furthermore, we also observed increased localization of FOXO3a in the nuclear fraction isolated from ULK1 KD HepG2 cells suggesting increased FOXO3a activity (Supplementary Figures S4C,D). Therefore, to test if FOXO3a activation is indeed involved in the down-regulation of SREBF2 levels in ULK1 KD cells, we performed a rescue experiment in which both ULK1 and FOXO3a were silenced. Our results showed that the loss of FOXO3a in cells, significantly rescued the suppressive effect of ULK1 KD on SREBF2 and its target transcriptional target HMGCR (Figures 4C,D). Intriguingly, as the rescue of HMGCR expression by co-silencing of FOXO3a and ULK1 was not complete, it is possible that other ULK1 regulated FOXO (Forkhead box) proteins such as FOXO1 (Koo et al., 2018) may also be involved in SREBF2 modulation and needs further investigation. The effects of ULK1 KD on AKT activation along with SREBF2 and HMGCR levels were also corroborated using a pharmacological inhibitor of ULK1, SBI-0206965, in HepG2 cells (Figure 4E). Therefore, these results establish a mechanistic link between ULK1 and SREBF2 via AKT-FOXO3a pathway (Figure 4F). However, the molecular basis of AKT regulation by ULK1 remains to be investigated.

## Discussion

The MVA pathway is the major metabolic pathway involved in sterol synthesis and has been extensively investigated for its role(s) in cardiovascular diseases. Statins are a very effective class of drugs that target HMGCR, a key rate limiting enzyme in cholesterol biosynthesis (Stancu and Sima, 2001) and are used to treat hypercholesterolemia. However, despite their effectiveness, some patients develop resistance to statins (Reiner, 2014). Given these issues, there have been considerable efforts to develop novel strategies which target cholesterol biosynthesis (Bou Malham and Goldberg, 2016; Sinha et al., 2019). Of note, the inhibition of MVA signaling has shown promising results in limiting cancer progression (Mullen et al., 2016) including liver cancers (Ogura et al., 2018). Interestingly, ULK1 inhibitors have also shown their efficacy in limiting cancer growth (Egan et al., 2015); thus, it is possible that some of the anti-tumor effects of ULK1 inhibitors are mediated through their effect on the MVA pathway.

Our results uncover a novel autophagy independent signaling by ULK1, which involves the regulation of anabolic AKT signaling and nuclear activity of FOXO transcription factor. Although the autophagy-lysosomal pathway is known to regulate nuclear receptor action (Siong Tan et al., 2019; Sinha et al., 2020), the direct effect of ULK1 on nuclear receptor/transcription factor activity remains less explored. As FOXO3a and related FOXO family proteins are involved in a myriad of cellular processes and human diseases (Lee and Dong, 2017), the modulation of ULK1 opens an exciting possibility of targeting FOXO related disorders.

Concerning the effect of ULK1 silencing on systemic cholesterol levels in mouse, we did not observe a significant reduction in serum total cholesterol albeit it showed a downward trend. We believe that this effect could be due to the transient nature of the knockdown experiments performed in this study. Perhaps, a chronic suppression using a liver-specific Ulk1 knockout model or pharmacological inhibitors would be needed to resolve it better. However, considering the functional redundancy of different ULK isoforms, the role of ULK2 in the regulation of cholesterogenic genes remains to be investigated. Although beyond the scope of the present study, further work using ULK1 inhibitors in animal models of hypercholesterolemia or carcinogenesis may be useful to test the translational value of ULK1 inhibitors, either alone or as an adjunct with statins, for the protection or treatment of diseases associated with deranged cholesterol synthesis.

## Materials and Methods

### Cell Culture

Mouse hepatocytes AML12 (CRL-2254) cells were maintained at 37°C in DMEM-F12 1:1 containing 10% fetal bovine serum (FBS), 1× ITS (Thermo Fisher Scientific, 41400), 10 nM dexamethasone and 1× penicillin/streptomycin, in a 5% CO_2_ humidified incubator. HepG2 cells were maintained at 37°C in DMEM containing 10% FBS. For siRNA transfection, cells were transfected using RNAiMAX (Thermo Fisher Scientific, 13778150) with *Ulk1* (Thermo Fisher Scientific, s75753), *Atg5* (Thermo Fisher Scientific, s62452), Becn1 (Thermo Fisher Scientific, s80166), *FoxO3a* (Cell Signaling Technology, #6302) siRNA (10 nM) for 48 or 72 h, followed by harvesting cells for RNA or protein extraction. ULK1 inhibitor SBI-0206965 (Selleckchem, Catalog No. S7885) was added at a concentration of 10 μM for 48 h.

### *In vivo* siRNA Administration to Liver

C57BL/6N male mice (4–6 weeks old) were administered 40 μg of *in vivo*-ready *Ulk1* (Thermo Fisher Scientific, s75753) or negative siRNA (Thermo Fisher Scientific, 4390843) every 24 h for 3 days via hydrodynamic tail-vein injection protocol using Mirus Bio TransIT-QR Delivery Solution as per manufacturer’s guidelines. All experiments were performed according to institutional animal ethics guidelines at Duke-NUS Medical School, Singapore.

### MicroArray and RNA-Seq Analysis

Gene expression microarray profiling was performed using GeneChip Mouse Gene 2.0 ST Array (Affymetrix) on pool of four samples. The cDNA generation, labeling and hybridization were performed at Duke-NUS Genome Biology Facility, Duke-NUS Graduate Medical School, Singapore. Gene expression signals were quantile normalized, and differentially expressed genes were identified via ANOVA models, using treatment specific contrasts (Partek Genomics Suite software, version 6.6). Statistical significance of differentially expressed genes was ascertained via the false discovery rate (FDR). RNA-seq analysis was performed on a pool of four samples per group. RNA-Seq Library Construction and Library QC, c-bot Cluster Generation and Hi-Seq3000 sequencing performed at Duke-NUS Genome Biology Facility, Duke-NUS Graduate Medical School, Singapore. Pathway enrichment analysis was conducted via the Gene Set Enrichment Analysis tool using a list of KEGG and Reactome pathways extracted from the Molecular Signatures Database^1^. Significance of pathway enrichment was ascertained by permutation testing of gene sets and calculation of FDR. The microarray and RNA-Seq data have been deposited to public databases with accession numbers GSE147579 and PRJNA625896, respectively.

### RNA Isolation and qRT-PCR

Total RNA was isolated and qRT-PCR was performed using the QuantiTect SYBR Green PCR Kit (Qiagen, 204141) according to manufacturer’s instructions. Gapdh gene was used for normalization. KiCqStart^®^ SYBR^®^ Green Primers were purchased from Sigma-Aldrich, United States. Primer ID were: Mouse Srebf2 (M_Srebf2_1), Human Srebf2 (H_SREPF2_1), Mouse Hmgcs1 (M_Hmgcs1_1), Human Hmgcs1 (H_HMGCS1_1), Mouse Hmgcr (M_Hmgcr_1), Human Hmgcr (H_HMGCR_1), Mouse Mvk (M_Mvk_1), Human Mvk (H_MVK_1), Mouse Pmvk (M_Pmvk_1), Mouse Mvd (M_Mvd_1), Human Mvd (H_MVD_1), Mouse Fdps (M_Fdps_1), Mouse Sqle (M_Sqle_1), Human Sqle (H_SQLE_1), Mouse Lss (M_Lss_1), Human Lss (H_LSS_1), Mouse Dhcr7 (M_Dhcr7_1), Human Dhcr7 (H_DHCR7_1), Mouse Gapdh (M_Gapdh_1), Human Gapdh (H_GAPDH_1).

### Cholesterol Measurement

Intracellular cholesterol levels in AML-12 cells were visualized using Filipin staining and quantitation was performed using Image J software. Four equal sized areas with 20 cells each were chosen in each image and the fluorescence intensity of each area was determined. These values were then used to calculate the average fluorescence of each image and then of control (set to 100%) and Ulk1 silenced group. Mouse liver total cholesterol was measured using commercial kit (MAK043, SIGMA-ALDRICH, United States).

### Western Blotting

Cells or tissue samples were lysed using CelLytic^TM^ M Cell Lysis Reagent (Sigma, C2978) and immunoblotting was performed as per manufacturer’s guidelines (Bio-Rad Laboratories, United States). Nuclear fractionation was done using NE-PER^TM^ Nuclear and Cytoplasmic Extraction Reagents (Thermo Fisher Scientific, 78833). Image acquisition was done using ChemiDoc (Bio-Rad ChemiDoc^TM^ MP System, 1708280). Densitometry analysis was performed using ImageJ software (NIH, Bethesda, MD, United States). Antibodies used were anti-ULK1 (Cell Signaling Technology, #8054), anti-GAPDH (Cell Signaling Technology, #5174), anti-phospho AKT (Cell Signaling Technology, #4060), anti-AKT (Cell Signaling Technology, #4685), anti-FoxO3a Ser^253^ (Cell Signaling Technology, #12829), anti-phospho FoxO3a (Cell Signaling Technology, #9466), anti-Beclin (Cell Signaling Technology, #3495), anti-ATG5 (Cell Signaling Technology, #12994), anti-SREBP2 (ABCAM, #ab30682), anti-HMGCR (ABCAM, #ab174830).

### Statistics

Results are expressed as mean ± SD. The statistical significance of differences was assessed by unpaired student *t*-test or one-way ANOVA when comparing different groups.

## Data Availability Statement

The datasets generated for this study can be found in the GSEA (GSE147579) and SRA (PRJNA625896).

## Ethics Statement

The animal study was reviewed and approved by IACUC, NUS.

## Author Contributions

RS conceived the experiments, researched the data and revised, wrote, and approved the manuscript. SG and PY researched the data and revised and approved the manuscript. SR, LI, JZ, NG, BA, and BS performed the experiments. All authors contributed to the article and approved the submitted version.

## Conflict of Interest

The authors declare that the research was conducted in the absence of any commercial or financial relationships that could be construed as a potential conflict of interest.

## Footnotes

1 http://software.broadinstitute.org/gsea/msigdb

## Abbreviations

ULK1: Unc-51 like autophagy activating kinase 1
SREBF2: sterol regulatory element binding factor 2
*Hmgcs1*: hydroxymethylglutaryl-CoA synthase 1
*Hmgcr*: 3-hydroxy-3-methylglutaryl-CoA reductase
*Mvk*: mevalonate kinase
*Pmvk*: phosphomevalonate kinase
*Mvd*: mevalonate diphosphate decarboxylase
*Lss*: lanosterol synthase
*Sqle*: squalene monooxygenase
*Dhcr7*: 7-dehydrocholesterol reductase
*Fdps*: farnesyl diphosphate synthase
FOXO3a: forkhead box O3a
NES: normalized enrichment score
NOM *p*-val: nominal *p* value.
