# Supplementary material for: Loss of ULK1 Attenuates Cholesterogenic Gene Expression in Mammalian Hepatic Cells
Source: Front Cell Dev Biol. 2020 Sep 30;8:523550. doi: 10.3389/fcell.2020.523550 (PMC7554540; doi:10.3389/fcell.2020.523550)
Supplement: Supplementary file 1 [file Data_Sheet_1.docx]

**Supplementary Tables**

**Suppl Table 1**: List of pathways effected by *Ulk1* silencing in AML-12 cells.

| NAME | PROBE | GENE SYMBOL | GENE_TITLE | RANK IN GENE LIST | RANK METRIC SCORE | RUNNING ES | CORE ENRICHMENT |
| --- | --- | --- | --- | --- | --- | --- | --- |
| row_0 | GGPS1 | null | null | 2382 | 0.125294 | -0.32155 | No |
| row_1 | EBP | null | null | 5169 | -0.11521 | -0.69951 | No |
| row_2 | DHCR24 | null | null | 5503 | -0.15306 | -0.7364 | No |
| row_3 | MVK | null | null | 7031 | -0.67988 | -0.90687 | Yes |
| row_4 | SC5DL | null | null | 7034 | -0.6872 | -0.86626 | Yes |
| row_5 | TM7SF2 | null | null | 7050 | -0.71186 | -0.82599 | Yes |
| row_6 | LBR | null | null | 7074 | -0.74681 | -0.78475 | Yes |
| row_7 | HMGCR | null | null | 7099 | -0.84142 | -0.73801 | Yes |
| row_8 | MSMO1 | null | null | 7115 | -0.909 | -0.68601 | Yes |
| row_9 | FDFT1 | null | null | 7122 | -0.92628 | -0.63174 | Yes |
| row_10 | PMVK | null | null | 7125 | -0.95276 | -0.57534 | Yes |
| row_11 | HMGCS1 | null | null | 7151 | -1.03224 | -0.51739 | Yes |
| row_12 | DHCR7 | null | null | 7183 | -1.15986 | -0.45268 | Yes |
| row_13 | MVD | null | null | 7184 | -1.16041 | -0.38366 | Yes |
| row_14 | SQLE | null | null | 7186 | -1.1695 | -0.31423 | Yes |
| row_15 | NSDHL | null | null | 7197 | -1.2219 | -0.24293 | Yes |
| row_16 | FDPS | null | null | 7230 | -1.35925 | -0.16649 | Yes |
| row_17 | LSS | null | null | 7238 | -1.41442 | -0.08333 | Yes |
| row_18 | CYP51A1 | null | null | 7240 | -1.44494 | 0.002486 | Yes |

**Suppl Table 2**: Gene set enrichment in Cholesterol biosynthesis pathway involving *Ulk1* silencing in AML-12 cells*.* The highlighted genes were validated by qRT-PCR.

**Suppl Table 3**: List of pathways effected by *Ulk1* silencing in mouse liver.

| NAME | PROBE | GENE SYMBOL | GENE_TITLE | RANK IN GENE LIST | RANK METRIC SCORE | RUNNING ES | CORE ENRICHMENT |
| --- | --- | --- | --- | --- | --- | --- | --- |
| row_0 | GGPS1 | null | null | 435 | 0.325679 | -0.04612 | No |
| row_1 | DHCR24 | null | null | 4198 | -0.09913 | -0.59916 | No |
| row_2 | EBP | null | null | 4379 | -0.11557 | -0.61933 | No |
| row_3 | SC5DL | null | null | 6241 | -0.40001 | -0.873 | No |
| row_4 | LBR | null | null | 6480 | -0.52495 | -0.87857 | Yes |
| row_5 | DHCR7 | null | null | 6631 | -0.72162 | -0.85991 | Yes |
| row_6 | TM7SF2 | null | null | 6650 | -0.75478 | -0.81976 | Yes |
| row_7 | MSMO1 | null | null | 6683 | -0.86415 | -0.7755 | Yes |
| row_8 | FDFT1 | null | null | 6690 | -0.90143 | -0.72526 | Yes |
| row_9 | MVK | null | null | 6698 | -0.95379 | -0.67219 | Yes |
| row_10 | HMGCR | null | null | 6711 | -1.03486 | -0.61527 | Yes |
| row_11 | LSS | null | null | 6722 | -1.09681 | -0.55454 | Yes |
| row_12 | PMVK | null | null | 6725 | -1.12803 | -0.49085 | Yes |
| row_13 | MVD | null | null | 6729 | -1.16187 | -0.42539 | Yes |
| row_14 | CYP51A1 | null | null | 6730 | -1.18639 | -0.35809 | Yes |
| row_15 | NSDHL | null | null | 6743 | -1.42748 | -0.2789 | Yes |
| row_16 | FDPS | null | null | 6746 | -1.4662 | -0.19602 | Yes |
| row_17 | HMGCS1 | null | null | 6747 | -1.47335 | -0.11245 | Yes |
| row_18 | SQLE | null | null | 6751 | -1.99278 | 1.48E-04 | Yes |

**Suppl Table 4**: Gene set enrichment in Cholesterol biosynthesis pathway involving *Ulk1* silencing in mouse liver*.* The highlighted genes were validated by qRT-PCR.

**Suppl Table 5**: List of pathways effected by *Ulk1* silencing in HepG2 cells (RNA-Seq data set).

| NAME | PROBE | GENE SYMBOL | GENE_TITLE | RANK IN GENE LIST | RANK METRIC SCORE | RUNNING ES | CORE ENRICHMENT |
| --- | --- | --- | --- | --- | --- | --- | --- |
| row_0 | GGPS1 | null | null | 2137 | 0.153342 | -0.25102 | No |
| row_1 | CYP51A1 | null | null | 2852 | -0.07486 | -0.29572 | No |
| row_2 | MSMO1 | null | null | 4885 | -0.6913 | -0.53152 | Yes |
| row_3 | FDPS | null | null | 5085 | -0.7661 | -0.50155 | Yes |
| row_4 | LBR | null | null | 5690 | -1.04279 | -0.5303 | Yes |
| row_5 | FDFT1 | null | null | 5976 | -1.20676 | -0.5128 | Yes |
| row_6 | MVK | null | null | 6042 | -1.24559 | -0.4634 | Yes |
| row_7 | SQLE | null | null | 6067 | -1.26514 | -0.40805 | Yes |
| row_8 | NSDHL | null | null | 6103 | -1.28368 | -0.35431 | Yes |
| row_9 | HMGCR | null | null | 6338 | -1.45716 | -0.32941 | Yes |
| row_10 | TM7SF2 | null | null | 6359 | -1.4703 | -0.27349 | Yes |
| row_11 | EBP | null | null | 6374 | -1.48227 | -0.21669 | Yes |
| row_12 | DHCR24 | null | null | 6380 | -1.48872 | -0.15859 | Yes |
| row_13 | LSS | null | null | 6596 | -1.74838 | -0.13094 | Yes |
| row_14 | HSD17B7 | null | null | 6712 | -1.98168 | -0.08879 | Yes |
| row_15 | HMGCS1 | null | null | 6719 | -1.98933 | -0.03084 | Yes |
| row_16 | IDI1 | null | null | 6802 | -2.21056 | 0.016094 | Yes |

**Suppl Table 6**: Gene set enrichment in Cholesterol biosynthesis pathway involving *Ulk1* silencing in HepG2 cells (RNA-Seq)*.* The highlighted genes were validated by qRT-PCR.

| NAME | PROBE | GENE SYMBOL | GENE_TITLE | RANK IN GENE LIST | RANK METRIC SCORE | RUNNING ES | CORE ENRICHMENT |
| --- | --- | --- | --- | --- | --- | --- | --- |
| row_0 | LDLR | null | null | 211 | 1.937882 | 0.040849 | No |
| row_1 | ACAT1 | null | null | 3615 | -0.30176 | -0.38091 | No |
| row_2 | FDPS | null | null | 5085 | -0.7661 | -0.52238 | Yes |
| row_3 | ACSS2 | null | null | 5310 | -0.85374 | -0.48342 | Yes |
| row_4 | ACAT2 | null | null | 5785 | -1.09314 | -0.48068 | Yes |
| row_5 | FDFT1 | null | null | 5976 | -1.20676 | -0.43679 | Yes |
| row_6 | MVK | null | null | 6042 | -1.24559 | -0.37478 | Yes |
| row_7 | SQLE | null | null | 6067 | -1.26514 | -0.30683 | Yes |
| row_8 | NSDHL | null | null | 6103 | -1.28368 | -0.24048 | Yes |
| row_9 | HMGCR | null | null | 6338 | -1.45716 | -0.20296 | Yes |
| row_10 | TM7SF2 | null | null | 6359 | -1.4703 | -0.13443 | Yes |
| row_11 | DHCR24 | null | null | 6380 | -1.48872 | -0.0659 | Yes |
| row_12 | LSS | null | null | 6596 | -1.74838 | -0.02563 | Yes |
| row_13 | HMGCS1 | null | null | 6719 | -1.98933 | 0.028116 | Yes |

**Suppl Table 7**: Gene set enrichment in CUSTOM_SREBP2_DSIL pathway involving *Ulk1* kd in HepG2 cells (RNA-Seq)*.*
