# Supplementary material for: Loss of ULK1 Attenuates Cholesterogenic Gene Expression in Mammalian Hepatic Cells
Source: Front Cell Dev Biol. 2020 Sep 30;8:523550. doi: 10.3389/fcell.2020.523550 (PMC7554540; doi:10.3389/fcell.2020.523550)
Supplement: Supplementary file 2 [file Data_Sheet_2.docx]

**Supplementary Figures
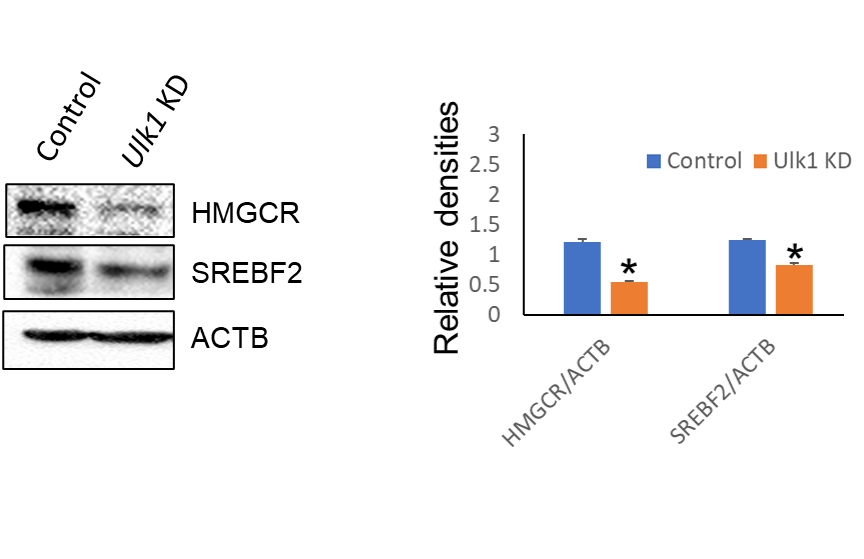
**

**Suppl Figure 1. Silencing of of ULK1 down-regulate the expression of cholesterol biogenesis proteins *in vivo*.** Representative immunoblot showing the protein levels of HMGCR and SREBP2 in mouse liver treated with ± *Ulk1* siRNA (40ug/72 h). Values are means ±SD (n=3,*p<0.05).

**
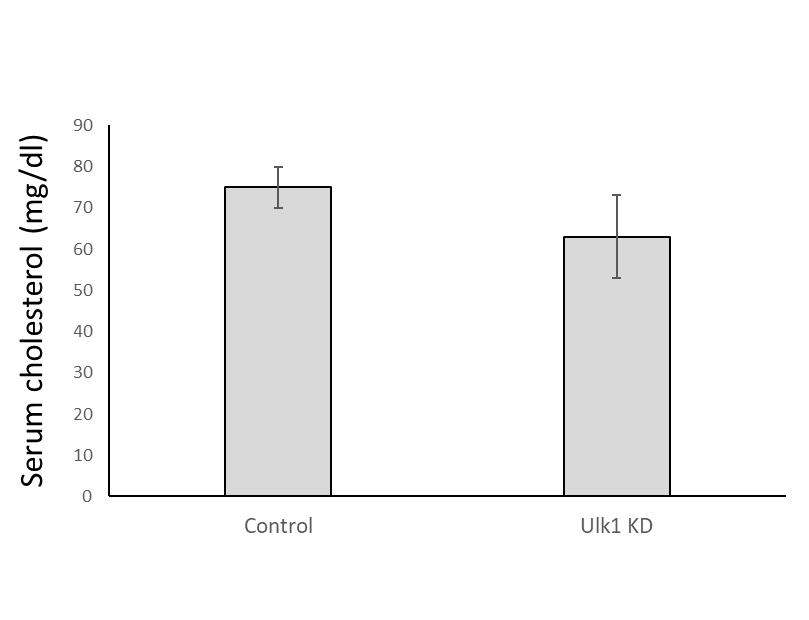
**

**Suppl Figure 2. Effect of Ulk1 silencing on systemic circulating cholesterol levels *in vivo*.** Histogram showing serum cholesterol levels in mouse liver treated with ± *Ulk1* siRNA (40ug/72 h). Values are means ±SD (n=3).

**
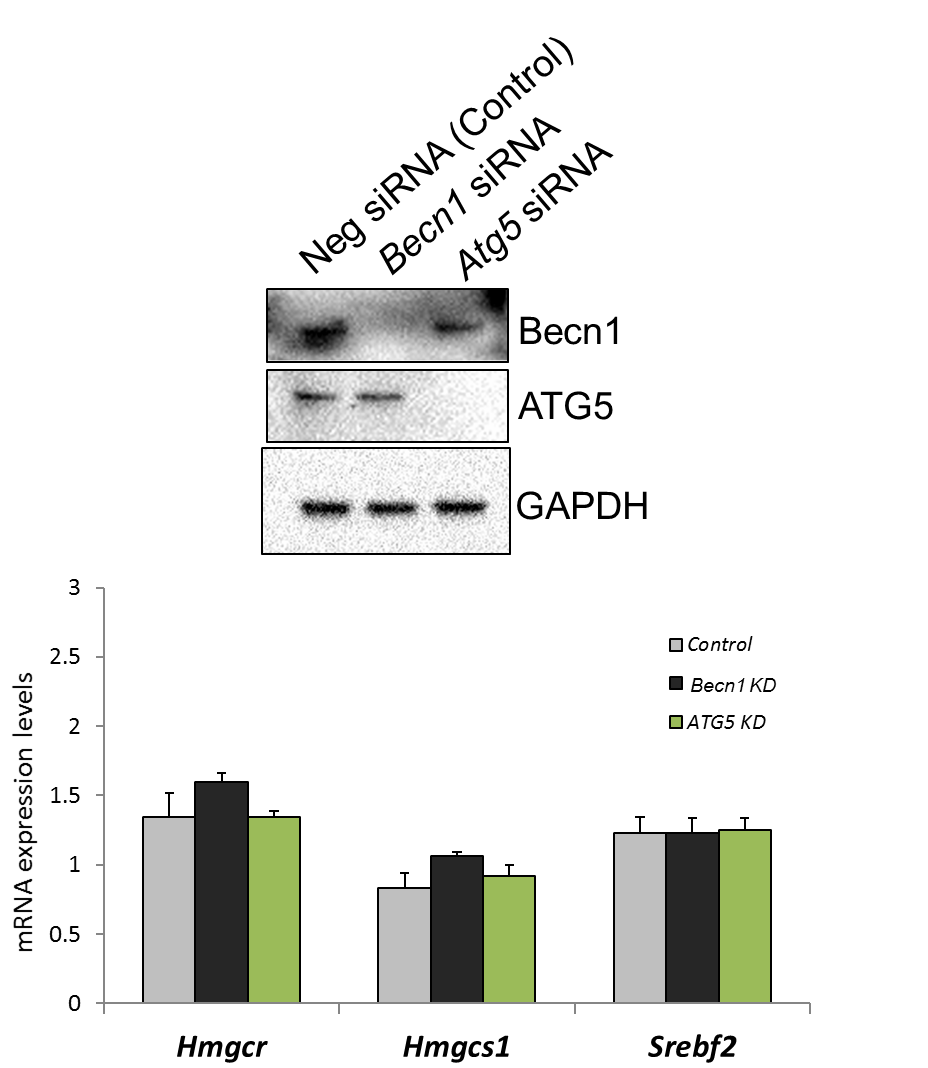
**

**Suppl Figure 3. Loss of ATG5 and Becn1 does not alter cholesterol biogenesis gene expression in AML-12 cells.** qRT-PCR validation of cholesterol biosynthesis genes enriched in the pathway analysis treated with ATG5 (ThermoFisher Scientific*,* s62452) and Becn1 (ThermoFisher Scientific, s80166) siRNA’s at a dose of 10nM for 72hrs (n=3,*p<0.05).

**
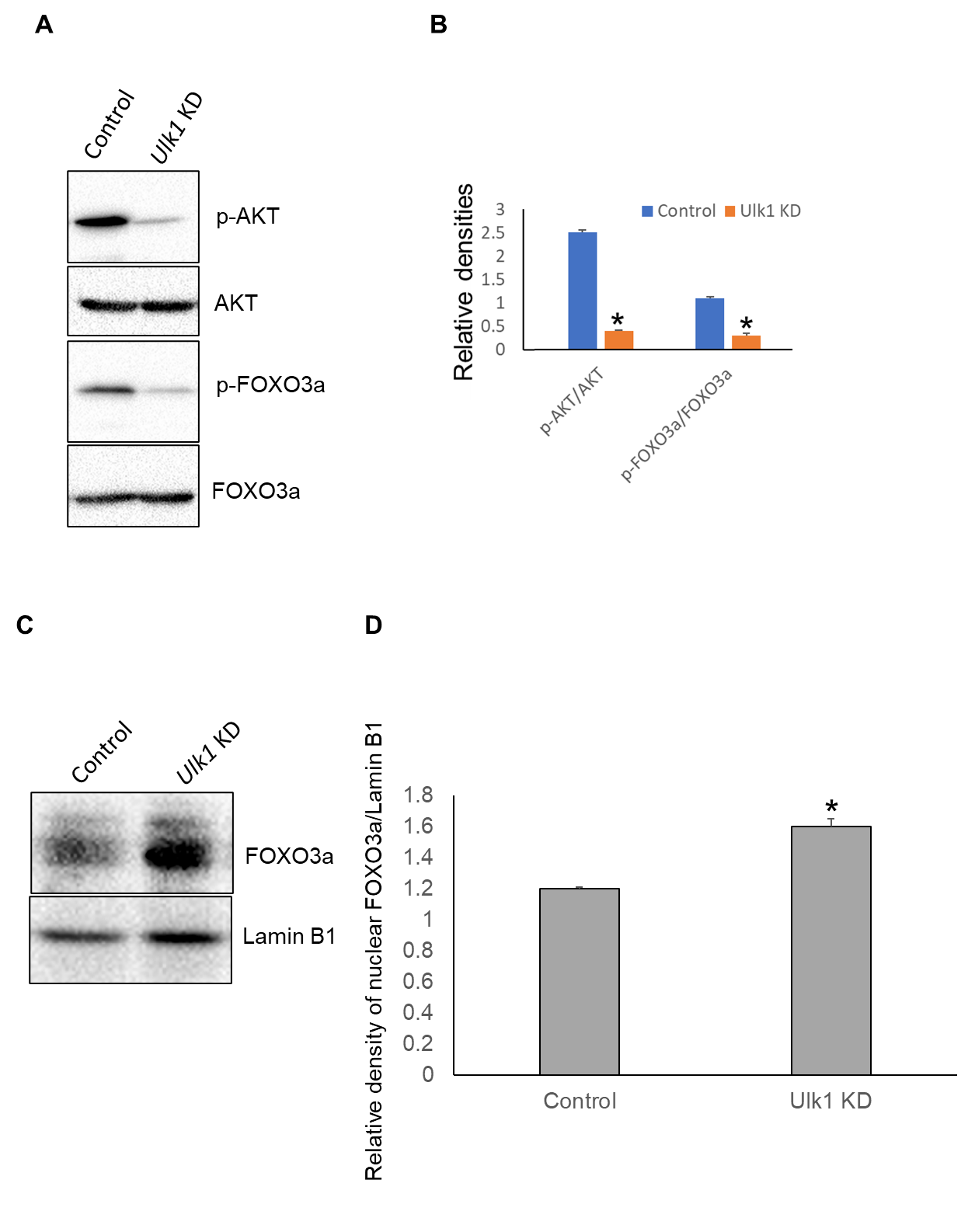
**

**Suppl Figure 4. Effect of ULK1 silencing on AKT-FOXO3a signalling. (A, B)** Representative immunoblot showing the protein levels of phospho and total AKT and FOXO3a in mouse liver treated with ± *Ulk1* siRNA (40ug/72 h). Values are means ±SD (n=3,*p<0.05). **(C, D)** Representative immunoblot and desitometric analysis showing increased levels of FOXO3a in the nuclear fraction of HepG2 cells treated with *Ulk1* siRNA at a dose of 10nM for 48hrs (n=3,*p<0.05).
